# Comparative Metabolomic Sampling of Upper and Lower Airways by Four Different Methods to Identify Biochemicals That May Support Bacterial Growth

**DOI:** 10.3389/fcimb.2018.00432

**Published:** 2018-12-18

**Authors:** Hugo Farne, Helen T. Groves, Simren K. Gill, Isobel Stokes, Scott McCulloch, Edward Karoly, Maria-Belen Trujillo-Torralbo, Sebastian L. Johnston, Patrick Mallia, John S. Tregoning

**Affiliations:** ^1^COPD and Asthma, National Heart and Lung Institute, Imperial College London, London, United Kingdom; ^2^Mucosal Infection and Immunity, Section of Virology, Imperial College London, London, United Kingdom; ^3^School of Veterinary Medicine, Faculty of Health & Medical Sciences, University of Surrey, Guildford, United Kingdom; ^4^Metabolon, Durham, NC, United States

**Keywords:** airway metabolome, airway sampling methods, healthy volunteers, upper airways, lower airways, synthetic adsorptive matrix strips

## Abstract

Bacteria need nutrients from the host environment to survive, yet we know little about which biochemicals are present in the airways (the metabolome), which of these biochemicals are essential for bacterial growth and how they change with airway disease. The aims of this pilot study were to develop and compare methodologies for sampling the upper and lower airway metabolomes and to identify biochemicals present in the airways that could potentially support bacterial growth. Eight healthy human volunteers were sampled by four methods: two standard approaches - nasal lavage and induced sputum, and two using a novel platform, synthetic adsorptive matrix (SAM) strips—nasosorption and bronchosorption. Collected samples were analyzed by Ultrahigh Performance Liquid Chromatography-Tandem Mass Spectroscopy (UPLC-MS/MS). Five hundred and eighty-one biochemicals were recovered from the airways belonging to a range of metabolomic super-pathways. We observed significant differences between the sampling approaches. Significantly more biochemicals were recovered when SAM strips were used, compared to standard sampling techniques. A range of biochemicals that could support bacterial growth were detected in the different samples. This work demonstrates for the first time that SAM strips are a highly effective method for sampling the airway metabolome. This work will assist further studies to understand how changes in the airway metabolome affect bacterial infection in patients with underlying airway disease.

## Introduction

Extracellular bacterial pathogens in the airways require biochemicals from the airway environment to grow: indeed Louis Pasteur described the body as a culture vessel as early as the 1870s (Brown et al., 2008). Understanding more about these biochemicals will help the development of new strategies to treat and prevent bacterial infections. In order to further our understanding of the biochemicals present in the airways, the optimum sampling methods and sites need to be determined. Metabolomics is a new, rapidly expanding field of systems biology that involves the measurement of all small biologic molecules, chemicals and metabolites in a biological sample. These molecules are a distinct class of compounds from nucleic acids or proteins and therefore metabolomics complements the other “omics” i.e., transcriptomics, genomics, and proteomics. Because the metabolome is more dynamic than the transcriptome or proteome, metabolomics has the ability to detect rapid changes resulting from acute pathological or environmental events. This makes it especially attractive for studying the airways as they are exposed to frequently changing environmental conditions and acute insults.

The study of the respiratory metabolome is still in its infancy and studies have focused mainly on the identification of potential disease and pathogen biomarkers. Studies investigating the airway metabolome have used different collection techniques including exhaled breath condensate, sputum, and bronchoalveolar lavage (BAL) (Nobakht et al., 2015), all of which have advantages and disadvantages. Exhaled breath condensate and sputum are simple and non-invasive but can be affected by a wide range of factors including equipment, sample preparation, food and drink, salivary contamination, and environmental temperature and humidity (Prieto et al., 2007; Czebe et al., 2008; Kullmann et al., 2008a,b). BAL is invasive and suffers from excessive and variable dilution (Leaker et al., 2015). More reproducible, ideally non-invasive, methods are needed to obtain samples for analyzing the airway metabolome. One approach that has led to a high yield of soluble immune mediators from the upper and lower airways is the use of synthetic adsorptive matrix (SAM) strips (Alam et al., 1992; Leaker et al., 2015). These strips have been used successfully to measure cytokines (Dhariwal et al., 2015; Leaker et al., 2015), viral RNA (Thwaites et al., 2017) and antibodies (Bergin et al., 2016; de Silva et al., 2017) but not, to date, to measure biochemicals. SAM strips have the advantage of avoiding the dilution that occurs with other sampling methods and can be used to sample both the upper and lower airways.

The aim of this pilot study was to compare the biochemical profile of four different methods of airway sample acquisition, two from the upper airway—nasal lavage and nasosorption SAM strips, and two from the lower airway—induced sputum and bronchosorption SAM strips. The samples were analyzed by Ultrahigh Performance Liquid Chromatography-Tandem Mass Spectroscopy (UPLC MS/MS). Of interest was whether biochemicals that support bacterial growth are present in the airways.

## Materials and Methods

### Study Participants

The study participants were healthy individuals between the ages of 18 and 55 years with no evidence of respiratory tract infection within the last 8 weeks (Table 1). The study was performed in accordance with relevant UK NHS Health Research Authority guidelines and regulations and received ethical approval (research ethics committee number 15/LO/0356 by London—Riverside Research Ethics Committee, UK) and informed written consent was obtained from all subjects.

**Table 1:** Volunteer baseline characteristics.

**ID**	**Sedation**	**Age**	**Gender**	**Serum glucose**
HF1	Midazolam	38	M	4.9
HF2	Midazolam	26	F	4.6
HF3	Midazolam	28	M	4.9
HF4	Midazolam	29	F	5.6
HF5	Midazolam	26	F	4.2
HF6	None	45	M	4.9
HF7	None	37	M	5.5
HF8	None	34	F	5.1

### Sampling Methods

All subjects underwent nasosorption, nasal lavage and bronchosorption sampling (the latter during bronchoscopy) at the same timepoint after 7 h of fasting. Induced sputum was collected 24 h prior to the bronchoscopy for safety reasons. Bronchoscopies (via the mouth) were performed in the Endoscopy Unit at St Mary's Hospital, in accordance with British Thoracic Society guidelines (Du Rand et al., 2013) and previous studies (Footitt et al., 2016). Subjects were offered sedation with intravenous midazolam (2–10 mg as necessary) and topical lidocaine was used for local anesthesia which was not expected to change the recovered metabolome, applied to the upper and lower airways.

#### Nasal Lavage

Five milliliter of sterile 0.9% saline was introduced into the left nostril via a 10 ml syringe attached to a disposable hollow nasal adapter—single nozzle, 19 mm nasal “olives” (Hunt Developments, Midhurst, West Sussex, UK), used to obstruct the nostril and prevent leakage, and saline was then withdrawn and flushed back into the nasal cavity 20 times over 1 min. The fluid was collected with, no further processing, in a sterile pot and stored at −80°C.

#### Nasosorption™

Performed as described (Chawes et al., 2010) and demonstrated (Thwaites et al., 2018) previously, prior to nasal lavage to avoid contamination with saline. Strips of a hydrophilic polyester synthetic absorptive matrix (SAM) measuring 7 × 35 mm (Hunt Developments, UK) were inserted gently into the nasal cavity under direct vision laterally against the anterior portion of the inferior turbinate. A nasal clip was applied, and the SAM was left for absorption for 2 min. The strips were then placed in an Eppendorf tube and immediately frozen at −80°C.

#### Bronchosorption™

The bronchosorption™ device (supplied as individual packaged single-use sterile medical devices: Hunt Developments) was passed down the operating port of the bronchoscope as demonstrated (Thwaites et al., 2018). The distal end of the inner probe incorporates a folded strip of SAM measuring 1.8 × 30 mm which is placed on the bronchial mucosa for 30 s, and then withdrawn back into its sheath before the complete device is removed from the bronchoscope. The sampling end of the probe was cut off, placed in an Eppendorf tube and immediately frozen at −80°C.

#### Induced Sputum

Three percentage of hypertonic saline was administered via ultrasonic nebuliser for 2 min periods with monitoring of FEV_1_ as per previous studies (Mallia et al., 2012) until a suitable sample was collected. Sputum samples were selected from saliva and immediately frozen at −80°C, without processing.

### Metabolomics

Once collected, samples were shipped to Metabolon (Durham, NC, USA) where they were processed, analyzed, and annotated. The analysis was essentially as described previously (Evans et al., 2014) with modifications as discussed below.

#### Sample Preparation

Samples were prepared using the automated MicroLab STAR® system. For liquid samples (sputum and nasal lavage), 100 μl fluid was added directly to 550 μl HPLC grade methanol (100%). SAM strips were taken from −80°C and incubated on ice overnight in 80% methanol. The methanol contained four recovery standards (DL-2-fluorophenylglycine, tridecanoic acid, d6-cholesterol and 4-chlorophenylalanine) to allow confirmation of extraction efficiency. Proteins were precipitated after vigorous shaking for 2 min (Glen Mills GenoGrinder 2000) followed by centrifugation, then divided equally into four equal aliquots (85 μl each). Samples were placed briefly on a TurboVap® (Zymark) 40°C for 90 min to remove organic solvent. Two aliquots of each sample were reconstituted in 50 μl of 6.5 mM ammonium bicarbonate in water (pH 8) for the negative ion analysis and another two aliquots of each were reconstituted using 50 μl 0.1% formic acid in water (pH ~3.5) for the positive ion method. A cocktail of internal standards were spiked into every analyzed sample (in the reconstitution buffer), allowing instrument performance monitoring and aided chromatographic alignment. The standards added at extraction and reconstitution steps are listed in the Biological Samples: Supplementary Information 2 of Evans et al. (2014).

#### Ultrahigh Performance Liquid Chromatography-Tandem Mass Spectroscopy (UPLC-MS/MS)

All methods utilized a Waters ACQUITY UPLC and a Thermo Scientific Q-Exactive high resolution/accurate mass spectrometer (MS) interfaced with a heated electrospray ionization source and Orbitrap mass analyser operated at 35,000 mass resolution. Four UPLC methods were used for the different compound types, as described in detail in Evans et al. (2014). The dried sample extract aliquots were reconstituted in 85 μl buffer compatible to each of the four methods: two aliquots in 6.5 mM ammonium bicarbonate in water (pH 8) for negative ion analysis; two aliquots in 0.1% formic acid in water (pH ~3.5) for positive ion analysis. For more hydrophilic compounds, the extract was gradient eluted from a C18 column (Waters UPLC BEH C18-2.1 × 100 mm, 1.7 μm) using water and methanol, containing 0.05% perfluoropentanoic acid and 0.1% formic acid. For more hydrophobic compounds, the extract was gradient eluted from the same C18 column using methanol, acetonitrile, water, 0.05% perfluoropentanoic and 0.01% formic acid and operated at an overall higher organic content. Another aliquot was analyzed using basic negative ion optimized conditions using a separate dedicated C18 column. The basic extracts were gradient eluted from the column using methanol and water, however with 6.5 mM Ammonium Bicarbonate at pH 8. The fourth aliquot was analyzed via negative ionization following elution from a HILIC column (Waters UPLC BEH Amide 2.1 × 150 mm, 1.7 μm) using a gradient consisting of water and acetonitrile with 10 mM Ammonium Formate, pH 10.8. The MS analysis alternated between MS and data-dependent MS^n^ scans using dynamic exclusion. MS and MS^n^ settings as described in Evans et al. (2014) Supplementary Figure 4. The scan range was 70–1000 *m/z* with a scan speed of ~9 scans *per second* (alternating between MS and MS/MS scans), and the resolution was set to 35,000 (measured at 200 *m/z)*. Mass calibration was performed as needed to maintain < 5 ppm mass error for all standards monitored.

#### Data Extraction and Compound Identification

Raw data was extracted, peak-identified and QC processed (Supplementary Data Sheet 2). Compounds were identified by comparison to Metabolon's library based on authenticated standards that contains the retention time/index (RI), mass to charge ratio (*m/z)*, and chromatographic data (including MS/MS spectral data) on all molecules present in the library (Dehaven et al., 2010; Evans et al., 2012). Biochemical identifications were based on three criteria: retention index within a narrow RI window of the proposed identification, accurate mass match to the library ± 10 ppm, and the MS/MS forward and reverse scores based on a comparison of the ions present in the experimental spectrum to the library spectrum. More than 4,500 commercially available purified standard compounds have been acquired and registered into this library for analysis on all platforms. Peaks were quantified using area-under-the-curve. When a biochemical was detected on more than one column, the highest quality data (most well-defined peak, clearest signal-to-noise ratio) was used for all samples.

### Biochemical Data Analysis

Area under the curve for peaks detected during UPLC were extracted using an in-house custom program (Metabolon). Included in the curation process to verify compound identity is a QC check of the peaks to verify the area under the curve calculations are correct (Evans et al., 2014). Those values are considered to be the “raw data” referred to as “Original Scale.” Original Scale data are median scaled on a per biochemical basis, and then any missing values are imputed with the sample set minimum, also on a per biochemical basis.

### Phenotype MicroArray™ (Biolog)

*Pseudomonas aeruginosa* (strain PAO1) was plated on LB agar and incubated overnight at 37°C. Colonies were scraped and resuspended in sterile water and IF-0a media and PM1/PM2 specific dye (Biolog, USA) as described previously (Lei and Bochner, 2013). Aliquots of cultures were placed into pre-coated PM1 or PM2 plates and incubated in an Omnilog machine (IBioIC, UK) for analysis over a period of 48 h. Analysis was performed by following the Biolog Data File Converter and Parametric Software.

### Bioinformatics and Statistical Analysis

For statistical analysis data were log transformed prior to calculations. The fold change values between groups were calculated using non log transformed data. Each raw biochemical value was scaled to set the median to 1 and missing values were replaced with the minimum scaled value. Two forms of unsupervised cluster analysis were used to visualize overall airway metabolomic profiles; Non-metric multidimensional scaling (NMDS) of the data using the Bray-Curtis dissimilarity index was performed in R version 3.2.3 (R Core Team, 2015) using the Vegan package (Dixon, 2003) and Principle component analysis (PCA). Hierarchical clustering of the data was performed in R using agglomeration strategy with average linkage and the Canberra distance metric which has been suggested to be one of the most suitable distance measures for metabolomic data analysis (Dixon et al., 2009) and has previously been used to perform hierarchical clustering of patient metabolomic profiles (Hadi et al., 2017). Pairwise *t*-tests with weighting of the false discovery rate were performed for each analyte between each sampling method. For grouped analysis, analysis of Similarity (ANOSIM) was used to determine whether metabolite composition of samples was significantly different both between and within sample sites. Pathway enrichment values for metabolomic sub-pathways were calculated for each pair-wise comparison as follows: the number of experimentally regulated metabolites in the sub-pathway (k) relative to the total number of detected metabolites in the sub-pathway (m) compared to the total number of experimentally regulated metabolites in that pair-wise comparison (n) relative to all detected metabolites in the study (N). Pathway enrichment = (k/m)/(n/M). Metabolomic networks were created using Cytoscape (Shannon et al., 2003) and pairwise comparisons of biochemicals significantly different in abundance (experimentally regulated) (*p* ≤ 0.05) were made between relevant sampling methods (matched pairs *t*-test). The size of the node in the network corresponds approximately to the size of the fold change in biochemical abundance between two sampling methods. Venn diagrams were generated using Venny 2.1 (Oliveros, 2007–2015).

### Data Availability

Raw MS plots are available at https://www.ebi.ac.uk/metabolights/MTBLS179; analyzed data is available as Supplementary Data Sheet 1.

## Results

### Biochemical Abundance Is Affected by Sampling Method and Site Sampled at the Global Level

The aim of the study was to determine the best sampling technique/collection substrate for the measurement of metabolites in the airways and to identify whether biochemicals that could support bacterial growth were present in the airway metabolome. Eight healthy subjects were recruited (baseline characteristics shown in Table 1) and provided one sample for each sampling method (nasosorption, nasal lavage, bronchosorption, and induced sputum) which covered two sampling sites, upper and lower airways. Global biochemical profiles were determined in the recovered samples and a total of 581 compounds of known identity (named biochemicals) were detected in the recovered samples. Since Lidocaine was detected in bronchosorption strips at three log_10_ greater abundance than in other sampling methods, reflecting its use during bronchoscopy, we removed it from the global analysis.

**Figure 1:**
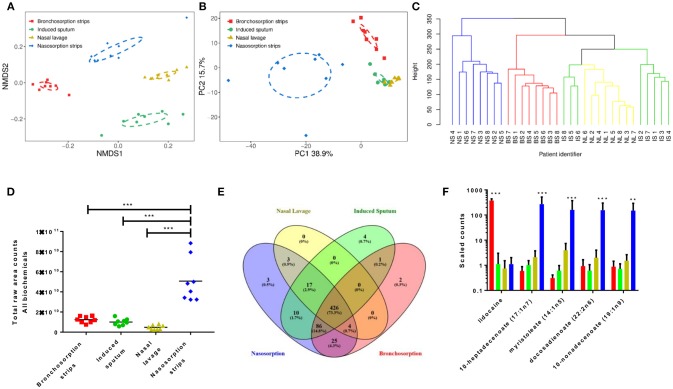
Different airway sampling techniques have distinct airway metabolomic profiles. Upper and lower airway surface liquids were sampled from eight healthy volunteers using bronchosorption strips (red), induced sputum (green), nasal lavage (yellow) and nasosorption strips (blue). Quantitative metabolic profiling by UPLC-MS/MS was performed on each airway sample, yielding 581 known compounds. Non-metric multidimensional scaling **(A)** and principle component analysis **(B)** were used on pre-scaled data to visualize the overall metabolomic composition of each airway sample. Hierarchical clustering analysis of similarities in metabolomic composition between individual samples and sampling sites (branches colored by sample site post clustering analysis) **(C)**. Total raw area counts for all detected biochemicals **(D)**. Venn diagram of individual biochemical identities by sampling method **(E)**. Individual analytes with highest variance between sampling methods **(F)** **p < 0.01 *** *p* < 0.001 between largest group and others. *n* = 8 volunteers.

We performed two types of unsupervised clustering analysis on the data to visualize the overall metabolomic profile at each sampling site; Non-Metric multi-dimensional Scaling (NMDS) and Principle Component Analysis (PCA), both of which compress multivariate data sets into two dimensions so overall trends can be more easily visualized and interpreted. Both are useful for interpreting data, NMDS analysis has the advantage of being able to use non-Euclidean distance measures as it aims to rank the pairwise dissimilarity between samples, whereas PCA aims to maximize the variance and so retains information on the magnitude of the distance between samples. NMDS analysis on the airway metabolome data resulted in four clusters, corresponding to each sampling method (Figure 1A), with clear, significant differences between the four sampling methods (p < 0.01) but little difference between subjects within each sampling method. The PCA revealed three distinct clusters; metabolomic profiles collected using naso and bronchosorption strips clustered separately from each other and from nasal lavage and induced sputum samples, which in turn clustered together (Figure 1B). The PCA did reveal a greater degree of variation with the SAM strips. Overall this indicates that the metabolites recovered by different sampling methods are significantly different.

To visualize the relationship between the individual samples and between the sampling sites in more depth, independent hierarchical clustering analysis using a bottom-up approach was performed. The branches were then colored according to the sample site (Figure 1C). Similarly to the NMDS and PCA analysis, individual samples were found to cluster predominantly within sampling site. Hierarchical clustering analysis further revealed that induced sputum samples and nasal lavage samples clustered more closely together, with some induced sputum samples clustering within the nasal lavage group, supporting the PCA results. These analyses suggest that the airway metabolome composition acquired using nasal lavage and induced sputum may be more similar than the NMDS analysis shows.

### Nasosorption Recovers the Highest Level of Biochemicals

Further analysis revealed that the nasosorption SAM strips recovered the most biochemicals, determined as total raw counts for all biochemicals (Figure 1D). This is most likely why the nasosorption strip samples clustered further away from other airway sampling methods in the hierarchical clustering analysis. Whilst 581 biochemicals were detected across all platforms, of these 426 were detected by all collection methods, most were found using more than one sampling method, only 9 were uniquely detected by a single method (Figure 1E). The unique biochemicals were N-acetyl-glucosamine 1-phosphate, N-ethylglycinexylidide (Bronchosorption); isovalerate, 2′-deoxycytidine 5′-monophosphate, 2′-deoxyadenosine 5′-monophosphate, 1,3-dimethylurate (Induced sputum) and vanillic alcohol sulfate, deoxycholate; chenodeoxycholate (Nasosorption): they did not belong to any specific families or pathways. We also looked to see if there were individual biochemicals with very high variance between the different sampling methods. Of the 5 biochemicals that had the highest variance, 4 were lipids and were significantly higher in the nasosorption strips than any other sample (Figure 1F).

**Figure 2:**
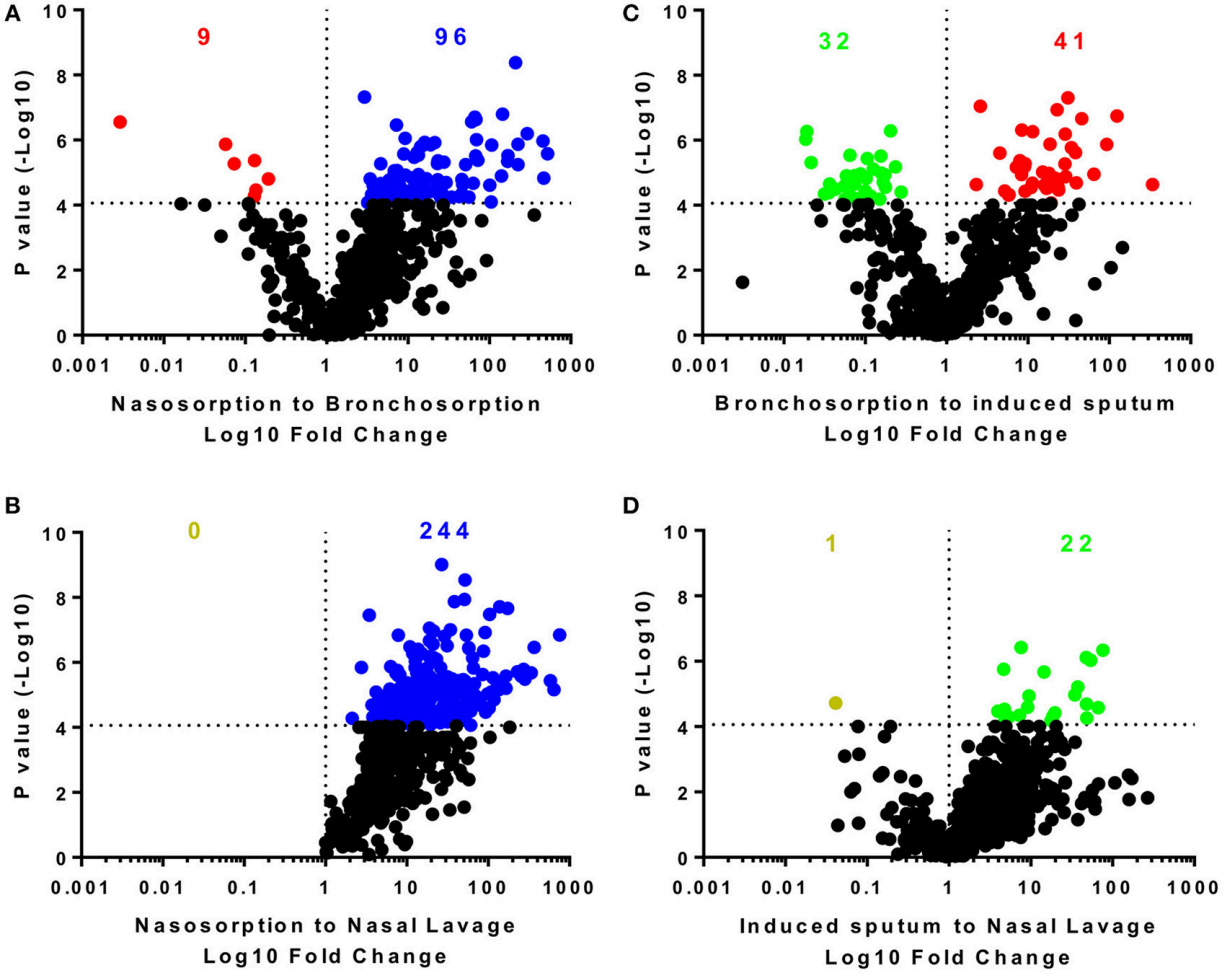
Distribution of individual biochemicals across sampling techniques. Pairwise comparisons of individual biochemicals were made for different sampling techniques either using similar methodologies **(A,D)** or sites **(B,C)**. Colored dots and numbers represent biochemicals significantly greater (blue nasosorption, red bronchosorption, green induced sputum, yellow nasal lavage) with a *p*-value measured by *t*-test cut off at 8 × 10^−5^ to reflect multiple testing.

We performed pairwise comparison on the different sampling methods (Figure 2): the nasosorption strips recovered the most biochemicals and at the highest level with 574 biochemicals detected in the nasosorption strips. Comparing nasosorption and bronchosorption, there were 96 biochemicals that were significantly more abundant in the nasosorption strips than the bronchosorption strips (Figure 2A). Of all sampling techniques, the fewest biochemicals were detected in the nasal lavage (Figure 2B). The profiles between the bronchosorption and induced sputum were slightly different (Figure 2C). There were also few differences between induced sputum and nasal lavage (Figure 2D). From this we conclude that the sampling method affects recovery and that SAM strips provide the greatest yield for both upper and lower airway metabolomic sampling.

### Biochemical Abundance Is Affected by Sampling Method and Site Sampled at the Super-Pathway and Individual Biochemical Level

**Figure 3:**
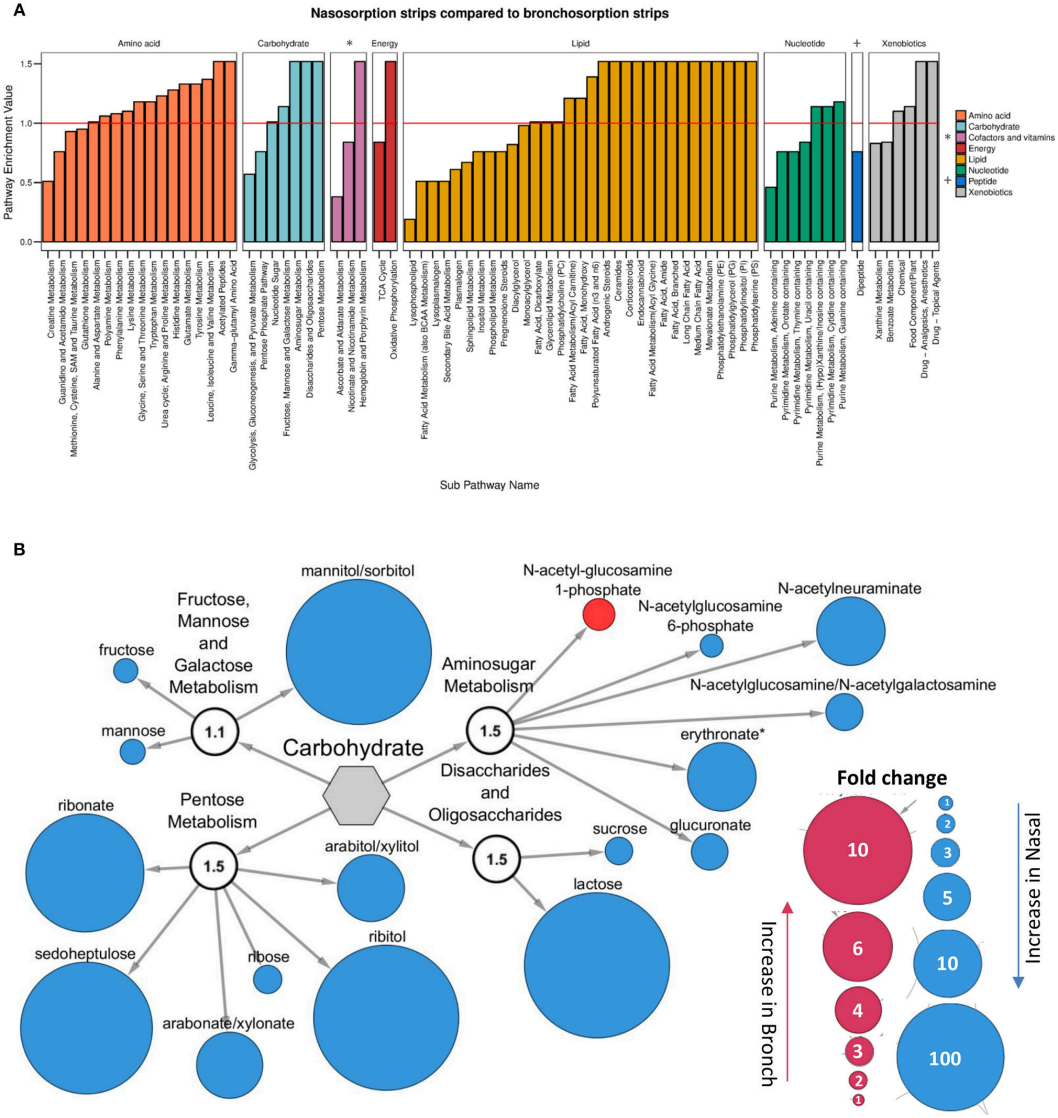
Analysis of nasosorption and bronchosorption data by super pathway. Pathway enrichment analysis comparing samples collected by nasosorption or bronchosorption. A pathway enrichment value of >1 indicates that this pathway contains more experimentally different metabolites relative to the study as a whole: red line indicates cut-off value of 1 **(A)**. Biochemicals in the enriched carbohydrate sub-pathways (pathway enrichment value displayed in white sub-family node) that were significantly higher (*p* ≤ 0.05) in nasosorption strips are shown in blue and significantly higher in bronchosorption strips in red **(B)**. Size of node is proportional to size of fold change. Analysis of *n* = 8 individual donors. Gray central node represents the metabolic carbohydrate superfamily.

Since the nasosorption and bronchosorption strips recovered the greatest amount of biochemicals from the upper and lower airways, respectively, we performed more detailed comparative analysis on these datasets. When analyzed at the level of KEGG super-pathways, the biochemicals detected can be split into amino acids (149 biochemicals), carbohydrates (33 biochemicals), cofactors and vitamins (19 biochemicals), energy (10 biochemicals), lipids (242 biochemicals), nucleotides (51 biochemicals), peptides (28 biochemicals), and xenobiotics (49 biochemicals). Pathway enrichment analysis enables the functional grouping of biochemicals; we used this approach to compare which sub-pathways contained the most significantly different biochemicals when comparing nasosorption and bronchosorption. Of the 74 sub-pathways that the biochemicals were grouped in, 42 were enriched (pathway enrichment value >1.0) for metabolites with significantly different abundance when comparing nasosorption to bronchosorption (Figure 3A).

We further investigated individual biochemicals and sub-pathways within super-pathways associated with supporting bacterial growth, including the carbohydrate and amino acid super-pathways (Krismer et al., 2014). Within the carbohydrate super-pathway, four sub-pathways were enriched: looking at individual members of these sub-pathways, there were 16 biochemicals that were significantly greater in nasosorption samples and one that was greater in bronchosorption strips (Figure 3B). A similar pattern was seen in the amino acid pathway (Figure S1). This may be reflective of differences between upper and lower airway metabolomes however some of the differences seen may be a consequence of the different sampling times between the nasosorption and bronchosorption strips.

### Differences in Upper and Lower Airway Metabolomes may Affect Bacterial Growth at These Sites

**Figure 4:**
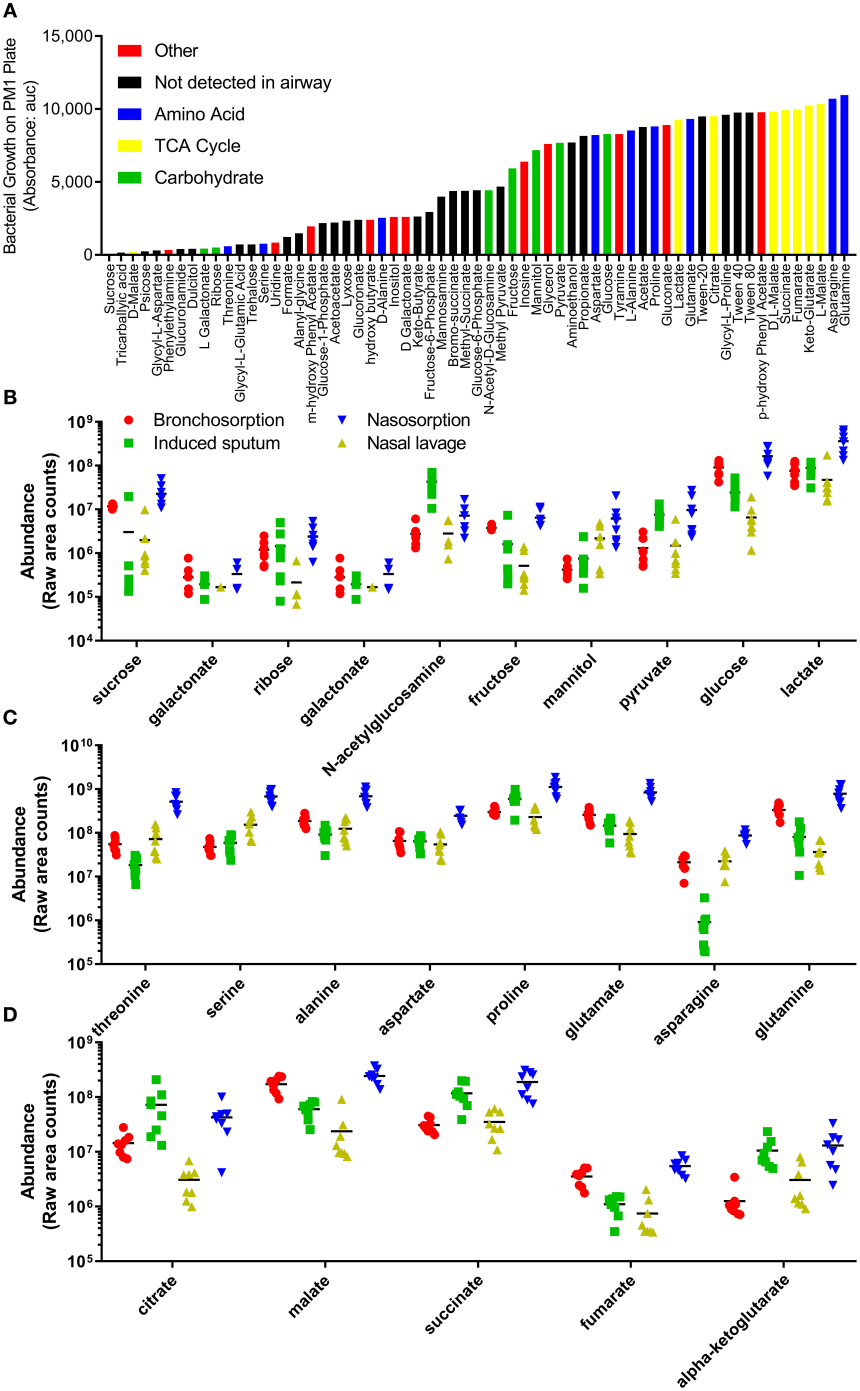
Profile of individual biochemicals that may support bacterial growth in the airways. Quantification of 48 h *P. aeruginosa* growth by Biolog phenotype microarray, black bars not detected in airway metabolome, colored bars represent biochemicals detected in airway, grouped by class **(A)**. Comparison of relative levels of individual biochemicals identified as supporting *P. aeruginosa* growth, collected using bronchosorption strips (red), induced sputum (green), nasal lavage (yellow) and nasosorption strips (blue), grouped by carbohydrates **(B)**, amino acids **(C)**, and TCA cycle **(D)**.

The underpinning aim of developing this methodology was to enable future studies investigating how changes in metabolites in airway disease could influence bacterial colonization and infection. To identify biochemicals that could potentially support bacterial growth, we used a commercially available Phenotype MicroArray™ (Biolog) (Bochner et al., 2001). As this was an approach to identify a range of biochemicals that could support growth, we investigated growth of the non-fastidious bacterial pathogen *Pseudomonas aeruginosa*. Using this system, we identified 62 carbon sources that support the growth of *P. aeruginosa* (data from PM1 plate, Figure 4A). 23 of these were detectable in the airways representing a range of carbohydrates (Figure 4B), amino acids (Figure 4C) and TCA cycle (Figure 4D) constituents. There were a number of other biochemicals that can potentially serve as an energy source for bacterial growth in the airways (Figure S2A). Of note, there was a difference between growth on the L and D forms of the different chemicals, with the L form supporting more growth than the D form, but because mass spectrometry is destructive it cannot detect differences in biochemical chirality in the airways. Several bacteria require co-factors to grow and interestingly nicotinamide and heme were both detectable in airway samples (Figure S2B). Additionally all of the core 20 proteinogenic amino acids were detectable in all of the samples (Figure S2C).

## Discussion

This is the first study to perform comparative metabolomic sampling of the upper and lower airways by different methods in the same individuals. Our data suggests that the use of SAM strips leads to a greater recovery of biochemicals than the traditional methods of airway sampling of nasal lavage and sputum induction. We were able to detect a number of biochemicals which can support the growth of bacteria, these may have an impact on susceptibility to bacterial infection both in health and disease, though further studies comparing healthy individuals to those with underlying disease need to be performed.

There were significant differences in biochemical abundance comparing sampling methods. Nasosorption strips gave a greater yield than nasal lavage and individual biochemical abundance was significantly greater for 244 of the biochemicals measured. This undoubtedly reflects the dilution associated with nasal lavage that is avoided using the SAM method and confirms the superiority of this technique. SAM strips were first developed to sample the epithelial lining fluid in the nose for cytokines (Chawes et al., 2010; Nicholson et al., 2011; Dhariwal et al., 2015). Insertion of SAM strips through a bronchoscope has extended this technique to sampling the epithelial lining fluid of the lower airways for cytokines (Jackson et al., 2014), but this might be applicable to other analytes. This was the first study to use SAM strips to collect samples for metabolomic analysis. The high yield of metabolites recovered by SAM strips reflects our recent experience with detecting virus-specific antibody where the SAM strips have significantly more concentrated sample recovery (de Silva et al., 2017). Interestingly in another study viral loads in nasosorption were several fold lower than in nasopharyngeal aspirate (NPA), another dilutional technique with variable yield (Thwaites et al., 2017). However, unlike NPA nasosorption correlated to clinical markers, thus the concentration of compounds may not always be higher on SAM strips than traditional sampling techniques, but it is more likely to be clinically relevant. The differences seen between the lower airway sampling methods of bronchosorption and induced sputum may reflect sampling methodology or be driven by the different compositions of bronchial epithelial lining fluid and sputum. Epithelial lining fluid is mainly shaped by epithelial cells, whereas inflammatory cells and micro-organisms affect the composition of sputum (Esther et al., 2015). One limitation in the comparison is that the induced sputum was collected on a different day to the other samples, which were all collected on the same day.

The NMDS and PCA data suggests that the metabolomic profile might differ between the upper and lower airways and therefore that, despite the attractiveness of non-invasive upper airway sampling methods, local sampling would be necessary to determine an accurate metabolomic profile of specific sites within the airways. There were differences in sampling methodologies which affects our ability to draw firm conclusions: the area of the strips were different, with nasosorption considerably greater, the bronchosorption sampling time was shorter than the nasosorption which may affect sample recovery and a clip was used for nasosorption but not for bronchosorption which may alter contact with the airway.

The ability of bacteria to obtain nutrients from the host is a major determinant of infection (Brown et al., 2008). If there are differences between upper and lower airway metabolomes it may determine growth tropism of bacteria (Bassis et al., 2015). Similar to previous studies (Krismer et al., 2014), we were able to detect a large number of biochemicals that could support bacterial growth in the airways. It is of note that many of the common bacterial airway colonizers are fastidious in their requirements for *in vitro* growth and, unlike culture medium, biochemicals at the host surfaces replenish over time, supporting continual growth of the colonizing bacteria. We hypothesize that the most common airway colonizers have adapted to the nutrients available in the airways, possibly losing biosynthetic genes for commonly available amino acids giving them a fitness advantage. However, other pathogens, for example *P. aeruginosa*, are able to grow on a broader range of biochemicals; which may reflect that *Pseudomonas* is principally an environmental bacteria that opportunistically infects airways. Since it is not adapted to the airways, *Pseudomonas* requires a more favorable environment to cause infection than bacteria that are normally present in the airways, for example the altered mucus-rich environment in cystic fibrosis.

It seems likely that the airway metabolome is dysregulated in pulmonary diseases and this impacts disease pathogenesis and clinical manifestations. However, other than the specific example of *Pseudomonas* in cystic fibrosis there have been few studies of this field. Using both *in vitro* and *in vivo* models and clinical observational data, we and others have demonstrated that dysregulation of a single metabolite—glucose—impacts bacterial lung infection (Philips et al., 2005; Garnett et al., 2013; Gill et al., 2016) and we have recently observed that airway glucose is significantly higher in patients with chronic obstructive pulmonary disease (COPD) and is related to higher bacterial loads (Mallia et al., 2017). Glucose is not the only biochemical in the airways that can support bacterial growth; therefore it is likely that other biochemicals influence pulmonary infection. Studies have indicated that the respiratory metabolome is altered in pulmonary diseases such as COPD (De Laurentiis et al., 2008, 2013; Basanta et al., 2010; Esther et al., 2011, 2015; Nobakht et al., 2015), cystic fibrosis (Wolak et al., 2009; Montuschi et al., 2012; Yang et al., 2012), and asthma (Carraro et al., 2007). However, these studies have focussed on identifying disease biomarkers, rather than the potential relationships between the host metabolome and bacterial infection. If the availability of biochemicals in the airways that can support bacterial growth has an impact on susceptibility to infection, then potentially manipulation of the airway metabolome has antimicrobial potential.

This was a pilot study with the aim of optimizing sampling techniques in a small cohort of healthy volunteers before performing an invasive technique (bronchoscopy) in patients with airway disease. As such there were a number of limitations that would need to be addressed in a future study to understand how the airway metabolome changes in disease. Differences in the salt concentration in the samples coming from the nasal lavage (0.9% saline) and induced sputum (3% hypertonic saline) might alter the liquid chromatography part of the LC-MS. Using this approach it is not possible to tell the source of the biochemical, whether host or bacteria, follow up studies labeling input material are required to investigate this; however the source of the biochemical is a separate issue to the effect it has on colonization. The study was qualitative, not quantitative and also the abundance levels of individual biochemicals are derived from different columns and ionization modes so are not directly comparable. But using computational tools to process the large data sets from each volunteer, we have demonstrated that the SAM strips yield higher levels of most biochemicals. We have demonstrated that a comprehensive metabolic profile of the upper and lower airways can be obtained using SAM strips. These methods can be used to investigate dysregulation of the respiratory metabolome in pulmonary diseases and how this may relate to pulmonary infection.

## Ethics Statement

This study was carried out in accordance with relevant guidelines and regulations and the protocol was approved by Riverside Research Ethics Committee, London, UK (research ethics committee number 15/LO/0356). All subjects gave written informed consent in accordance with the Declaration of Helsinki.

## Author Contributions

HF, M-BT-T, and PM collected clinical samples. HG, SG, IS, SM, and EK analyzed data. SJ, PM, HF, and JT designed study. JT and PM wrote the manuscript. All authors revised the manuscript and approved the final version for submission for publication.

### Conflict of Interest Statement

EK and SM are employees of Metabolon, which was contracted to do the mass spec analysis. The remaining authors declare that the research was conducted in the absence of any commercial or financial relationships that could be construed as a potential conflict of interest.

## Supplementary Material

Figure S1 Differences in individual amino acids in enriched pathways. Biochemicals in the enriched amino acid sub-pathways (pathway enrichment value displayed in white sub-family node) that were significantly higher (*p* ≤ 0.05) in nasosorption strips are shown in blue and significantly higher in bronchosorption strips in red. Size of node is proportional to size of fold change. Analysis of *n* = 8 individual donors. Gray central node represents the metabolic amino acid superfamily.

Figure S2 Biochemicals in the airway that could support bacterial growth. Comparison of relative levels of individual biochemicals identified as supporting *P. aeruginosa* growth, collected using bronchosorption strips (red), induced sputum (green), nasal lavage (yellow) and nasosorption strips (blue), grouped by biochemicals identified on PM1 **(A)** co-factors **(B)** and proteinogenic amino acids **(C)**.

Supplementary Data Sheet 1 Complete data set.

Supplementary Data Sheet 2 Quality control information.
