# Supplementary figures and images for: Comparative Metabolomic Sampling of Upper and Lower Airways by Four Different Methods to Identify Biochemicals That May Support Bacterial Growth
Source: Front Cell Infect Microbiol. 2018 Dec 18;8:432. doi: 10.3389/fcimb.2018.00432 (PMC6305596; doi:10.3389/fcimb.2018.00432)

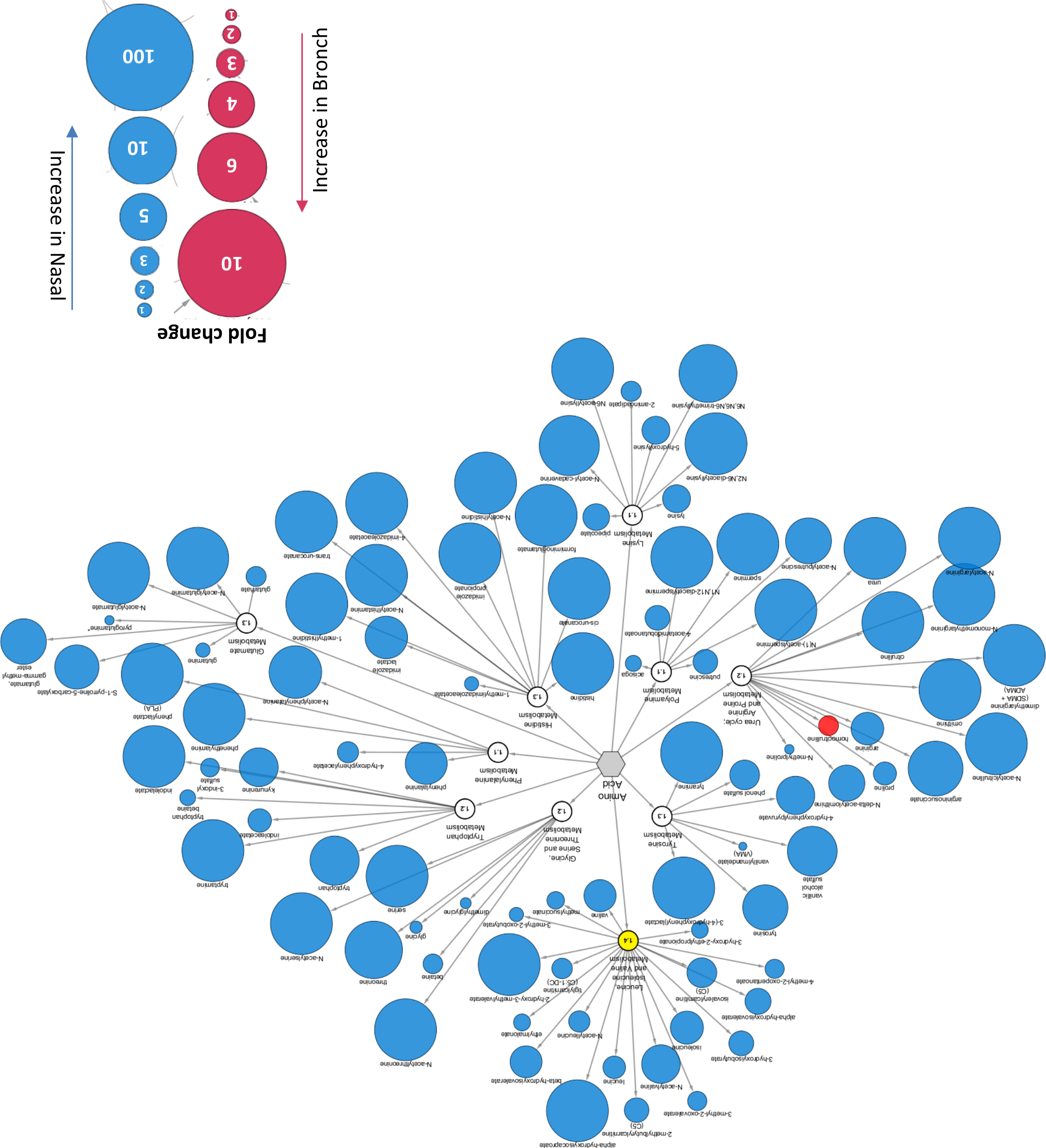

Supplement: Figure S1 — Differences in individual amino acids in enriched pathways. Biochemicals in the enriched amino acid sub-pathways (pathway enrichment value displayed in white sub-family node) that were significantly higher (p ≤ 0.05) in nasosorption strips are shown in blue and significantly higher in bronchosorption strips in red. Size of node is proportional to size of fold change. Analysis of n = 8 individual donors. Gray central node represents the metabolic amino acid superfamily. [file Image_1.TIF]

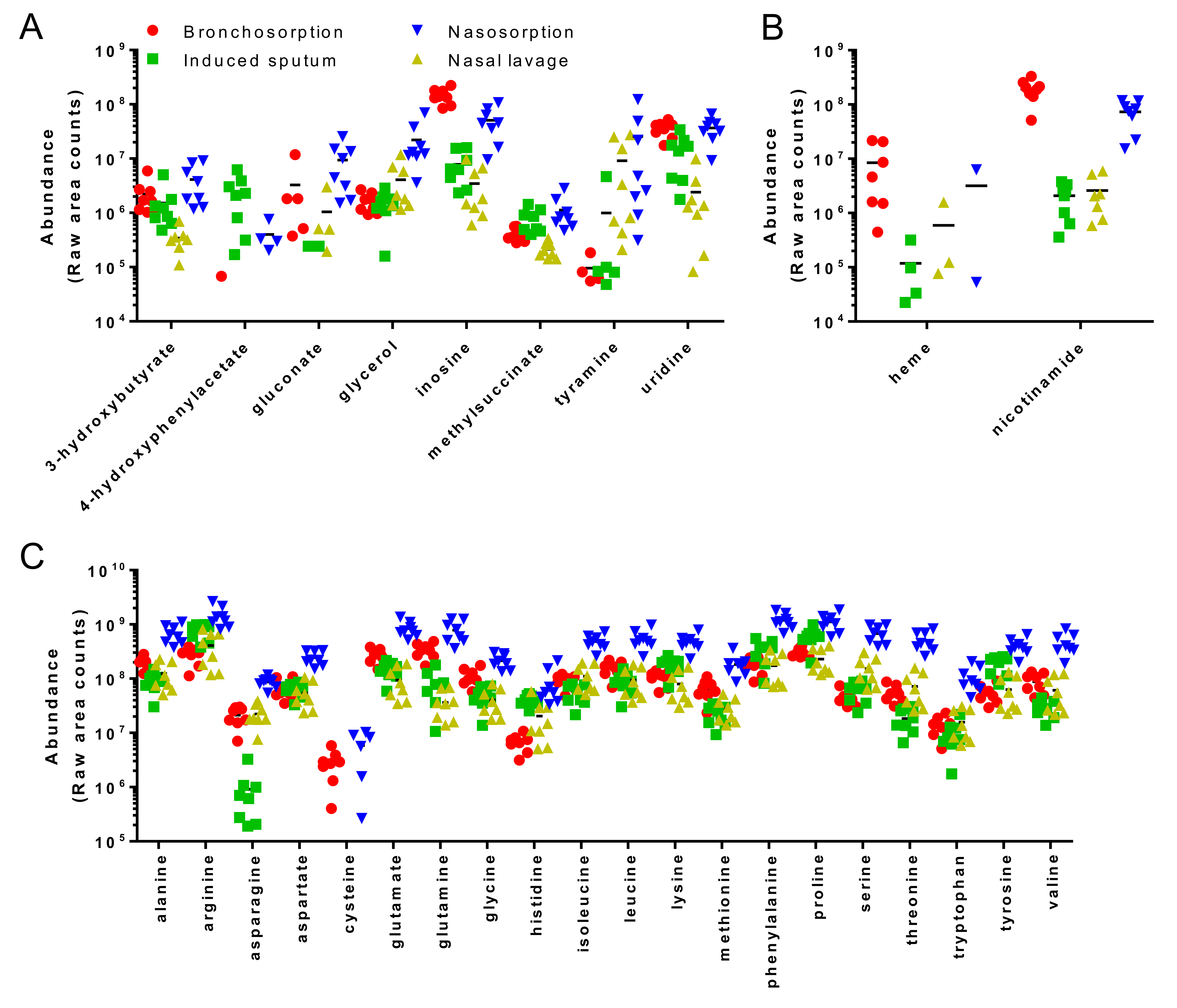

Supplement: Figure S2 — Biochemicals in the airway that could support bacterial growth. Comparison of relative levels of individual biochemicals identified as supporting P. aeruginosa growth, collected using bronchosorption strips (red), induced sputum (green), nasal lavage (yellow) and nasosorption strips (blue), grouped by biochemicals identified on PM1 (A) co-factors (B) and proteinogenic amino acids (C). [file Image_2.TIF]
